# Variable ability of rapid tests to detect *Mycobacterium tuberculosis rpoB* mutations conferring phenotypically occult rifampicin resistance

**DOI:** 10.1038/s41598-019-48401-z

**Published:** 2019-08-14

**Authors:** Gabriela Torrea, Kamela C. S. Ng, Armand Van Deun, Emmanuel André, Justine Kaisergruber, Willy Ssengooba, Christel Desmaretz, Siemon Gabriels, Michèle Driesen, Maren Diels, Sylvie Asnong, Kristina Fissette, Mourad Gumusboga, Leen Rigouts, Dissou Affolabi, Moses Joloba, Bouke C. De Jong

**Affiliations:** 1 Mycobacteriology Unit, Biomedical Sciences, Institute of Tropical Medicine, Antwerp, Belgium; 2 Laboratory of Clinical Bacteriology and Mycology, Department of Microbiology and Immunology, KU Leuven, Leuven, Belgium; 3 Cliniques Universitaires Saint-Luc, Brussels, Belgium; 4 Tuberculosis National Reference Laboratory, Cotonou, Benin; 5 Department of Medical Microbiology, College of Health Sciences Makerere University, Kampala, Uganda

**Keywords:** Microbiology, Infectious-disease diagnostics

## Abstract

We compared the ability of commercial and non-commercial, phenotypic and genotypic rapid drug susceptibility tests (DSTs) to detect rifampicin resistance (RR)-conferring ‘disputed’ mutations frequently missed by Mycobacterium Growth Indicator Tube (MGIT), namely L430P, D435Y, L452P, and I491F. Strains with mutation S450L served as positive control while wild-types were used as negative control. Of the 38 mutant strains, 5.7% were classified as RR by MGIT, 16.2% by Trek Sensititre MYCOTB MIC plate, 19.4% by resazurin microtiter plate assay (REMA), 50.0% by nitrate reductase assay (NRA), and 62.2% by microscopic observation direct susceptibility testing (MODS). Reducing MGIT rifampicin concentration to 0.5 µg/ml, and/or increasing incubation time, enhanced detection of disputed mutations from 5.7% to at least 65.7%, particularly for mutation I491F (from 0.0 to 75.0%). Compared with MGIT at standard pre-set time with 0.25 µg/ml ECOFF as breakpoint, we found a statistically significant increase in the ability of MGIT to resolve disputed mutants and WT strains at extended incubation period of 15 and 21 days, with 0.5 µg/ml and 1 µg/ml ECOFF respectively. MODS detected 75.0% of the I491F strains and NRA 62.5%, while it was predictably missed by all molecular assays. Xpert MTB/RIF, Xpert Ultra, and GenoscholarTB-NTM + MDRTB detected all mutations within the 81 bp RR determining region. Only GenoType MTBDR*plus* version 2 missed mutation L430P in 2 of 11 strains. Phenotypic and genotypic DSTs varied greatly in detecting occult rifampicin resistance. None of these methods detected all disputed mutations without misclassifying wild-type strains.

## Introduction

In the 2016 guidelines for the treatment of multidrug-resistant (MDR) tuberculosis (TB), WHO recommended the use of rapid drug susceptibility testing (DST) against rifampicin in adults and children^1,2^. Several rapid methods – both phenotypic and genotypic – were extensively evaluated, leading to their approval by WHO^3^. Among the phenotypic methods, the rapid automated BACTEC MGIT 960 DST SIRE method (MGIT) continues to be used due to a substantial gain in turnaround time compared to the standard DST on solid medium (Löwenstein Jensen, and Middlebrook 7H10 or 7H11 agar)^4,5^. Non-commercial DST methods, such as Microscopic Observation Drug Susceptibility Testing (MODS), Nitrate Reductase Assay (NRA) and Colorimetric Redox Indicator (CRI), have also been recommended for use at central reference laboratories^2,3^. To overcome limitations of phenotypic methods, such as turnaround time and high safety requirements, commercial molecular tests, namely Line Probe Assay (LPA) and the fully automated Xpert MTB/RIF (hereinafter referred to as classic Xpert) and Xpert MTB/RIF Ultra (hereinafter referred to as Ultra) were also endorsed by the WHO^6,7^. In contrast with the short conventional probes of classic Xpert, Ultra was designed with four long sloppy molecular beacon probes. The specific temperature at which the probe and amplicon hybrid denatures is recorded (melt peak temperature). The unique combination of melting temperature shift (∆Tm) – the difference between mutant and wild-type melt peak temperatures – and Ultra probe allows end-user to identify specific rifampicin resistance (RR)-conferring mutation^8^. Combinations of ∆Tm values and Ultra probes that unambiguously discriminate among RR-conferring mutations have been validated in a previous study^9^.

Discordances between phenotypic (in particular rapid liquid based, such as MGIT) and genotypic DSTs were largely attributable to occult resistance conferred by specific, uncommon *rpoB* mutations, referred to as ‘disputed’ mutations^10–12^. These strains were not rare among retreatment cases from two low-income settings in Africa and Asia^13^. They represented 23.4% (95% CI, 19.6–29.7) of RRDR mutants in new cases from recent random population surveys using molecular detection of rifampicin resistance in Bangladesh, Pakistan and Zimbabwe (own unpublished data). Although difficult to detect, they do cause resistance that is clinically important for the individual and for the community^11,14^. Moreover, they are equally likely to be associated with poor treatment outcome as the common *rpoB* mutations causing high-level rifampicin resistance^11,13,14^.

Miotto *et al*. hypothesized that disputed mutations reduce the affinity of rifampicin but do not completely prevent it from binding with the *rpoB* protein, supported by a molecular dynamics analysis that showed how H445 mutations still allow rifampicin to bind to the *rpoB* protein, albeit in a different conformation that allowed RNA synthesis to proceed, explaining partial inhibition on rifampicin-containing media^15^. Further, such strains may grow too slowly in DST due to loss of fitness^16,17^, although the drug-free growth controls are generally sufficiently well developed to validate the tests. Other rapid, growth-based DST techniques may thus also miss their resistance^18,19^. Although genotypic methods do not depend on growth, currently available commercial assays fail to detect mutations located outside the RRDR^20,21^.

MGIT uses pre-set standard conditions of rifampicin concentration based on the WHO critical concentration (CC) of 1 µg/ml^22^, inhibiting the growth of 99% of phenotypically wild-type (WT) strains, based on the proportion method that detects 1% of minority resistant strains, and pre-set incubation time, both of which were validated in several studies^23–25^. These conditions however are not optimal for detection of occult rifampicin resistance due to ‘disputed’ mutations, as the current CC of MGIT is above the ECOFF, resulting in a breakpoint artefact^26^. The minimum inhibitory concentration (MIC) of WT and mutant strains and their distribution are measures used to define the breakpoints to classify the susceptibility patterns of the strains. The MICs of WT strains overlap with those of mutant strains harbouring disputed mutations when pre-set standard MGIT conditions (incubation time and critical concentration of rifampicin) are employed. We thus explored whether extended incubation time in MGIT could reduce this overlap in MIC values between WT and mutant strains and compared the ability of pheno- and genotypic rapid tests, both commercial and non-commercial, to detect occult rifampicin resistance due to ‘disputed’ mutations.

## Materials and Methods

The panel consisted of clinical *Mycobacterium tuberculosis* (MTB) strains from the collection of the Supra-National TB Reference Laboratory (SRL) at ITM, Antwerp, partly from the Belgian Coordinated Collections of Microorganisms (BCCM/ITM)^27^. The strains harboured either the most common disputed *rpoB* mutations (n = 38; L430P [n = 11], D435Y [n = 10], L452P [n = 9] and I491F [n = 8])^28^, the most common high-level resistance mutation S450L as positive control (n = 5), or no mutation (WT) as negative controls (n = 13). These genotypes were determined by Sanger sequencing using primers for comprehensive amplification of the *rpoB* gene including cluster I (RRDR), cluster II (codons 490 and 491) and codons 170, 480, 552 and 592)^29^. The strains had been previously tested by the proportion method on Löwenstein-Jensen medium (LJ DST) with reading at 6 weeks incubation and standard 40 µg/ml critical concentration^30^. The WT reference strain *M*. *tuberculosis* H37Rv was tested by all the methods as quality control in each run. The tests were performed by experienced technicians blinded to previous and concurrent DST results, with each technician having a differently coded set of strains.

### Phenotypic drug susceptibility tests

The rapid phenotypic methods evaluated were: MGIT at 0.125, 0.25, 0.5 µg/ml of rifampicin and the standard critical concentration of 1 µg/ml with standard- as well as extended incubation time, NRA on Löwenstein-Jensen (LJ) medium, Resazurin Microtiter Assay (REMA), MODS and Trek Sensititre MYCOTB Minimum Inhibitory Concentration plate (TREK Diagnostic Systems, Cleveland, OH) referred to as MYCOTB.

For MGIT, MODS and MYCOTB, commercial media/kits were used following the supplier’s procedures, while the other DST methods and reagents were prepared in-house^31–33^. MYCOTB was performed in the Laboratory of Microbiology and Mycobacteriology in the Saint Luc University Hospital in Brussels because of its larger experience. All remaining phenotypic tests performed by qualified staff in Antwerp were set up from suspensions of the same freshly grown subculture.

MODS, NRA and REMA tests were performed as previously described using an undiluted McFarland 1 standard as the inoculum for NRA, and a 1/10 dilution for REMA. This dilution was also inoculated as NRA control^32,34^.

The breakpoints for determining the MIC were set at rifampicin 1 µg/ml for MYCOTB^33^ and 0.5 µg/ml for REMA^35^. The concentration of rifampicin used for MODS was 1 µg/ml^36,37^. The MIC was defined as the lowest concentration of drug that prevents any growth. Any strain was considered resistant when the MIC obtained was higher than the breakpoint specified for each method. Growth of MTB in MODS was declared only for well-developed micro-colonies, with cording. As per published guidelines of the MODS manufacturer, when a positive result (≥2 colonies) was observed in each of the two drug free wells, the well containing drug was examined the same day^37^. The absence of growth, or presence of a single Colony Forming Unit (CFU) in either of both control wells, was considered an invalid result. MODS tests were read at 5, 7, 9, 14 and 21 days until a resistant result was obtained otherwise the strain was declared sensitive. For MYCOTB, the growth of MTB was monitored after incubation at 35–37 °C for 10 days. If growth was poor the plates were re-incubated up to an additional 11 days. The reading was done using a manual viewer with final results reported when there was adequate growth in the drug-free control wells, otherwise the test was declared invalid. The MIC was defined as the lowest antibiotic concentration that inhibited visible growth.

In the REMA assay, resazurin was added after seven days and tests were read 2 days later. The MIC was defined as the lowest concentration of the drug that prevents any change of colour of the resazurin from blue to pink while those wells showing a purple colour were considered as inhibition of MTB growth.

A rifampicin 40 µg/ml critical concentration was used for the NRA as previously described. Final NRA readings were at 7, 10 or 14 days, depending on the positive reaction of a control tube^38^.

Any strain was considered resistant in MGIT960 when the Growth Units (GU) in the drug-containing tube were >100 at the moment that the GU in the Control tube reached 400, per manufacturer’s instructions. For the strains not yet considered resistant, the readings were continued until the GU in each of the drug-containing tubes reached >100, or until a maximum of 28 days, using BD software V3.05 A. The time to resistance was converted to the decimal fraction of the day by dividing hours by 24. Any invalid tests were repeated once before reporting the final results used for analysis.

### Genotypic drug susceptibility tests

The rapid genotypic methods evaluated were: GenoType MTBDR*plus* version 2, Hain (referred to as LPA-Hain) (lots #OV00096 and OV00098), Genoscholar TB-NTM + MDRTB, NIPRO (referred to as LPA-NIPRO, lot #16A00), Xpert MTB/RIF (G4 assay version 5, cartridge, lot #08806 and 09902), and Xpert MTB/RIF Ultra (assay version 2, cartridge lot #20502). These tests were performed according to the manufacturer’s instructions. RR-TB thermolysates for classic Xpert and Ultra were diluted as previously described to avoid too high a bacterial load, and achieve a bacilli concentration in the dynamic range of the assays^9,39^. For Ultra, the melting temperature (Tm) shift was calculated as a delta (Tm mutant – Tm wild-type)^9^.

### Analysis

The detection rate of the tests including the different MGIT conditions was calculated as the proportion of *M*. *tuberculosis* strains found resistant out of the mutant strains tested, with 95% confidence interval stratified by type and group of mutation (disputed versus undisputed). The proportion of WT strains testing rifampicin susceptible was used to evaluate specificity, and the reference standard was *rpoB* sequencing. The “N-1” Chi-squared test was used to calculate p-values^40,41^. The MIC values were calculated for the three conditions used in MGIT DSTs, the standard pre-set incubation time, extending the incubation to 15 days and to 21 days. The tentative ECOFF values used as breakpoint were calculated as the highest MIC value in µg/ml obtained for the WT strains tested in this study at each condition, as a proof of principle, in view of the low number of WT stains.

Permission from an ethics review board was not deemed necessary for this laboratory-based study on strains from a public culture collection, as the analysis did not include any patient related data.

## Results

Among the total of 56 strains tested, the following number of tests did not yield interpretable results after repeat testing, usually due to absence of growth of the controls: 3 (mutants) in MGIT, 3 (2 mutants and 1 WT) in REMA and 1 (WT) in MODS. One L452P mutant was not tested in MODS and MYCOTB. NRA did not yield any invalid or contaminated result.

The proportion of detected disputed rifampicin resistance varied widely between the different tests (Table 1); in descending order, MGIT (standard incubation time) 0.125 µg/ml (80.0%) >MODS (62.2%) >NRA (50.0%) >MGIT 0.25 µg/ml (31.4%) >REMA (19.4%) >MYCOTB (16.2%) >MGIT (standard incubation time) 0.5 µg/ml (14.3%) >MGIT (standard incubation time) 1.0 µg/ml (5.7%). However, specificity suffered for the most sensitive tests, with one (MODS) or two (MGIT 0.125) WT strains found resistant. Of the unmodified methods, MODS and NRA were the more sensitive methods- detecting ≥50% of the strains harbouring disputed mutations. Only NRA – without a false resistant result – can be considered accurate in this study. The results obtained by NRA were similar to those obtained previously by LJ DST (Table 1). MGIT 1.0 or 0.5 µg/ml and also MYCOTB detected less than 20% of the disputed mutations.

**Table 1:** Percentage of strains declared rifampicin resistant (mutants) and susceptible (WT) by five rapid phenotypic DST methods and the Proportion method (PM) on Löwenstein-Jensen (LJ).

*rpo*B sequencing result	(N)	BACTEC MGIT960 (standard incubation), %				REMA, %	MYCOTB, %	MODS, %	NRA, %	PM, %	p-value NRA vs PM
		0.125	0.25	0.5	1	0.5^a^	1^a^	1^a^	40^a^	40^a^	
L430P^b^	11	70.0	30.0	30.0	20.0	27.3	18.2	36.4	36.4	36.4	1.0000
D435Y^b^	10	88.9	55.6	11.1	0.0	11.1	30.0	80.0	60.0	70.0	0.6477
L452P^b^	9	75.0	12.5	0.0	0.0	12.5	0.0	62.5	44.4	66.7	0.3549
I491Fb^b^	8	87.5	25.0	12.5	0.0	25.0	12.5	75.0	62.5	75.0	0.6015
Overall detection of disputed mutations (C.I.)	38	80.0 (63.0–91.6)	31.4 (16.9–49.3)	14.3 (4.8–30.3)	5.7 (0.7–19.2)	19.4 (8.2–36.0)	16.2 (6.2–32.0)	62.2 (44.8–77.5)	50.0 (33.4–66.6)	60.5 (43.4–76.0)	0.3605
S450L^c^	5	100	100	100	100	100	100	100	100	100	NA
WT	13	84.6 (54.6–98.1)	100	100	100	100	100	91.7 (61.5–99.8)	100	100	NA

^a^Cut-off or critical concentration used to define resistance to rifampicin, in µg/ml.

^b^Disputed mutation.

^c^Non-disputed mutation.

WT: wild-type.

NA: Not applicable.

The detection rate varied by mutation. Less than 50% of L430P mutants were detected by any phenotypic method, except MGIT 0.125 µg/ml, whereas only MODS detected over 50% of L452P mutants (Table 1). Mutation D435Y was detected by MODS at 80.0% and by NRA at 60.0%, and mutation I491F by MODS at 75.0% and by NRA at 62.5%.

### Modification of MODS

Extending incubation of MODS up to a total of maximum 23 days resulted in all three L452P and one out of seven L430P strains correctly classified as resistant without affecting the classification of WT strains, although 2 strains each with the D435Y or I491F mutation remained false susceptible.

### Extension of MGIT reading time

Extending the incubation time in MGIT beyond 8 or 10 days after inoculation at rifampicin concentrations of 0.125 µg/ml and 0.25 µg/ml resulted in false resistance results among the wild-type strains after at least 9.0 days at 0.125 µg/ml and 10.2 days at 0.25 µg/ml (Table 2). At 0.5 µg/ml the reading could be extended to 15 days and at 1 µg/ml to 21 days, without resulting in false resistance.

**Table 2:** Range of time in days (minimum and maximum) of the automated reading for susceptible strains to be found resistant with extended incubation in MGIT.

*rpo*B sequencing result	Range of days in MGIT at critical concentration			
	0.125 µg/ml	0.25 µg/ml	0.5 µg/ml	1.0 µg/ml
L430P	10.4–13.3	8.0–21.7	10.9–27.5	8.9–>28.0
D435Y	>28.0	10.3–15.4	10.2–20.9	10.8–24.1
L452P	8.6–17.8	10.8–22.3	10.9–24.1	14.1–26.2
I491F	13.0	9.9–13.9	9.8–21.5	10.0–25.6
WT	9.0–23.1	10.2–15.8	16.3–>28.0	22.6–>28.0
H37Rv	10.9–>28.0	12.8–>28.0	17.5–>28.0	22.8–>28.0

H37Rv: *Mycobacterium tuberculosis* reference strain (ITM nr 083715).

With extended incubation in MGIT at different concentrations the detection rate of MGIT for disputed mutations increased significantly from 5.7% with the standard procedure (1 µg/ml, pre-set incubation time) to 68.6% using 1 µg/ml at 21 days (p < 0.0001), and to 65.7% using 0.5 µg/ml at 15 days (p < 0.0001) without misclassifying the WT strains (Table 3). Stratified by *rpoB* mutation, at 21 days using 1 µg/ml and at 15 days and 0.5 µg/ml, the sensitivity for the L430P mutation increased from 20.0% (standard conditions) to 50.0% (p = 0.1704). For L452P, undetected in standard MGIT, the detection reached 62.5% at 21 days (1 µg/ml) and at 15 days (0.5 µg/ml) (p = 0.0304). For D435Y, also undetected in standard MGIT, the detection increased to 88.9% (p = 0.0002) at 21 days (1 µg/ml) and 77.8% (p = 0.001) at 15 days (0.5 µg/ml). As for mutation I491F, none were detected using the standard MGIT procedure, whereas 75.0% were recognized as rifampicin resistant using either modification (p = 0.0027).

**Table 3:** Detection of disputed mutations by MGIT at standard and modified procedures (extended MGIT incubation period and reduced critical concentration to 0.5 µg/ml).

*rpo*B sequencing result	N	Standard condition						Extended incubation					
		CC 1 µg/ml at pre-set time			ECOFF 0.25 µg/ml			ECOFF 0.5 µg/ml 15 days			ECOFF 1 µg/ml 21 days		
		R	S	ND^a^	R	S	ND^a^	R	S	ND^a^	R	S	ND^a^
L430P	11	2	8	1	3	7	1	5	5	1	5	5	1
D435Y	10	0	9	1	5	4	1	7	2	1	8	1	1
L452P	9	0	8	1	1	7	1	5	3	1	5	3	1
I491F	8	0	8	0	2	6	0	6	2	0	6	2	0
Overall detection^b^ (%, CI 95%)		5.7 (0.7–19.2)			31.4 (16.9–49.3)			65.7 (47.8–80.9)			68.6 (50.7–83.2)		
p-value								vs ECOFF 0.25 µg/ml at standard incubation period: 0.0044			vs ECOFF 0.25 µg/ml at standard incubation period: 0.0020		
								ECOFF 0.5 µg/ml 15 days vs ECOFF 1 µg/ml 21 days: 0.7976					
S450L	5	5	0	0				5	0	0	5	0	0
WT	13	0	13	0				0	13	0	0	13	0
Detection of true WT (%)			100			100			100			100	

^a^ND: no data due to invalid result (due to no growth in the control tube).

^b^Rate of disputed mutations detected by each of the DST methods was calculated from the completed data excluding invalid results (due to no growth in the control tube).

**Figure 1:**
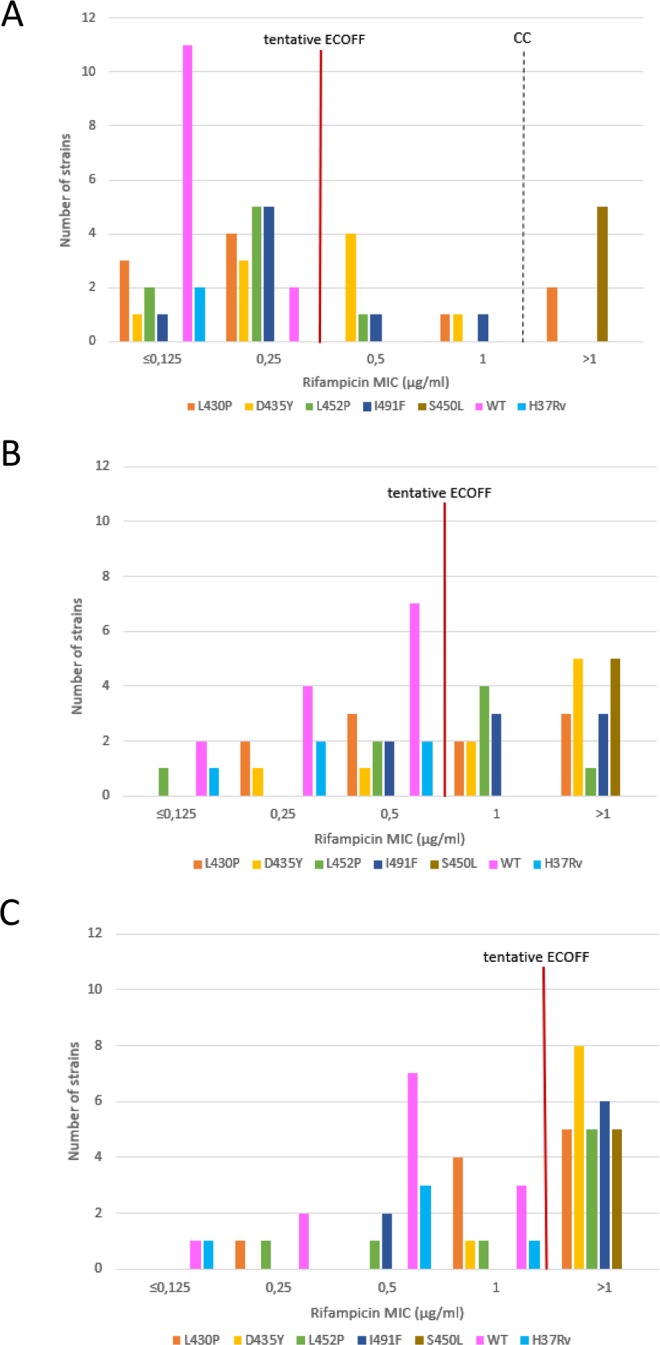
MIC distribution for the disputed rifampicin-resistant TB (RR-TB) strains at (**A**) standard MGIT conditions of 1 µg/ml rifampicin critical concentration at pre-set time; (**B**) 0.5 µg/ml rifampicin critical concentration at 15 days extended incubation; and (**C**) 1 µg/ml rifampicin critical concentration at 21 days extended incubation.

For MGIT, the distribution of MIC values for the mutant and WT strains were analysed using the tentative ECOFF values defined for each of the three conditions, 0.25 µg/ml for the standard pre-set time, 0.5 µg/ml for incubation at 15 days, and 1 µg/ml for the extension up to 21 days. Table S1 in supplement presents the MIC values for the three conditions, while Fig. 1A–C shows their distributions including the tentative ECOFFs. At standard conditions, using the CC of 1 µg/ml, only 2 (5.7%) out of 35 disputed mutant strains would be detected, whereas using the tentative ECOFF 0.25 µg/ml, 11 (31.4%) disputed mutants had MIC values higher than this breakpoint (p = 0.0060). At the modified MGIT conditions, extending the incubation period up to a total of 15 and 21 days, 23 (65.7%) and 24 (68.6%) MIC values of disputed mutants were significantly higher than the tentative ECOFFs (0.5 µg/ml and 1 µg/ml respectively). The difference between these two modifications was not significant (p = 0.7976). Detection of disputed mutants with these extended incubation periods however, was significantly better compared to the standard condition with 0.25 µg/ml tentative ECOFF (p = 0.0044 and p = 0.0020 for 15 days/0.5 µg/ml and 21 days/1 µg/ml respectively). All the WT strains had MIC values lower than or equal to the respective tentative ECOFFs at the three conditions evaluated (Tables 3 and S1).

### Genotypic drug susceptibility tests

All strains yielded interpretable results by the four methods evaluated (Table 4). Classic Xpert, Ultra, and LPA-NIPRO detected all the mutations in the RRDR. As expected, all four tests failed to detect all strains with the I491F mutation, located outside the RRDR. Only LPA-Hain missed two of eleven strains with mutation L430P. Overall detection rates of disputed mutations were thus 74% for LPA-Hain, and 79% for classic Xpert, Ultra, and LPA-NIPRO. The differences in the ability of these genotypic DSTs to detect disputed mutations were not statistically significant. All four genotypic DSTs correctly classified the WT strains.

**Table 4:** Detection of disputed mutations by genotypic methods.

*rpoB* sequencing result	N	LPA-Hain^a^		classic Xpert MTB/RIF		Xpert MTB/RIF Ultra		LPA-NIPRO^b^	
		R	S	R	S	R	S	R	S
L430P	11	9	2	11	0	11	0	11	0
D435Y	10	10	0	10	0	10	0	10	0
L452P	9	9	0	9	0	9	0	9	0
I491F	8	0	8	0	8	0	8	0	8
Total	38	28	10	30	8	30	8	30	8
Overall detection % (CI)		73.7 (0.60–0.88)		78.9 (0.66–0.92)		78.9 (0.66–0.92)		78.9 (0.66–0.92)	
S450L	5	5	0	5	0	5	0	5	0
WT	13	0	13	0	13	0	13	0	13

^a^LPA-Hain: GenoType MTBDR*plus* version 2.

^b^LPA-NIPRO: GenoscholarNTM + MDRTB II, NIPRO.

Table 5 shows classic Xpert and Ultra raw data observed for ‘disputed’ mutations L430P, D435Y, L452P, and I491F and undisputed mutation S450L. Classic Xpert probe reactions did not distinguish between ‘disputed’ (e.g. L452P) and undisputed (e.g. S450L) mutations situated in the same region covered by the probe. Ultra probes on the other hand, particularly rpoB4B and rpoB4A and corresponding ∆Tm values, were able to differentiate between L452P and S450L respectively.

**Table 5:** Comparison of classic Xpert and Ultra raw results.

*rpo*B sequencing result	N	classic Xpert	Xpert Ultra	
		Capturing probe	Capturing probe	∆Tm
L430P	11	missed probe A	rpoB1	5.8–6.3
D435Y	10	missed/delayed probe B	rpoB2	4.0–4.4
L452P	9	missed/delayed probe E	rpoB4B	5.7–6.1
I491F	8	ND	ND	ND
S450L	5	missed probe E	rpoB3	2.5–2.9
			rpoB4A	6.0–6.5

∆Tm: melting temperature shift between WT and mutant melt probes.

ND: strains that harbored corresponding mutation outside the RRDR yielded a “RIF Resistance Not Detected” result.

## Discussion

We studied a variety of rapid phenotypic and genotypic methods to detect rifampicin resistance by the four most prevalent disputed mutations frequently missed by phenotypic methods and almost systematically by automated MGIT960 – L430P, D435Y, L452P, and I491F^10^. None of the five rapid phenotypic DST methods evaluated - MGIT, REMA, MYCOTB, MODS and NRA – detected all strains harbouring these four mutations. The best balance between maximal detection of these mutants without risking false resistance was for NRA, with half of resistant strains correctly classified. This is comparable with the 60.5% detection by conventional LJ DST (Table 1), with the advantage that the turnaround time was substantially faster. MODS detected slightly more mutant strains (62.2%, non-significant), but misclassified one WT strain as resistant at as early as 7 days of incubation. The specificity of MODS for DST directly from sputum was previously reported to be reliable^42,43^. However, in this study, we performed indirect DST in which the inoculum was prepared from a solid culture isolate. Residual clumping of an insufficiently dispersed inoculum may have caused the false resistant result^42,43^. The remaining phenotypic methods detected ≤1/3 of the mutant strains.

Extending the MGIT incubation time increased the detection of disputed mutations, as is well known for LJ DST with final reading at 6 weeks, if not yet found resistant at 4 weeks^30^. Reportedly, rifampicin in 7H9 broth and LJ medium was nearly 50% degraded after one week at 37 °C, being a possible reason for false resistance categorization of *M*. *tuberculosis* isolates^44^. However, we did not detect false resistance at the six weeks reading on LJ, i.e. standard practice for the proportion method.

Also, lower rifampicin concentrations improved the detection of rifampicin resistance in MGIT. Although rifampicin at 0.125 µg/ml showed the highest overall MGIT detection rate, it revealed an unacceptable number of false resistant WT strains (‘true susceptible’ decreased to 85%).

The distributions of MIC values obtained at standard conditions revealed that the majority of the disputed mutants had a value below the tentative ECOFF previously reported^26,45^ and some of them were between the ECOFF and the critical concentration recommended by WHO and CLSI^46,47^, thus representing breakpoint artefacts. Lowering the CC to the tentative ECOFF (0.25 µg/ml) at standard incubation time, which is in line with the ECOFF previously reported^26^, allowed the detection of about one third of the disputed mutants. However, with 15 days extended incubation period at 0.5 µg/ml or 20 days at 1 µg/ml, the proportion of disputed mutants correctly classified as RR rose to two thirds, without misclassification of wildtype strains, in spite of concurrent increases in the MICs of WT and mutant strains. This delayed display of resistance might be explained by a fitness loss caused by these disputed mutations, yet this is difficult to distinguish from low level resistance allowing growth particularly at lower concentrations of the drug. Low level resistance is however unlikely to explain the general lack of sensitivity of MGIT at pre-set conditions of incubation time and concentration for these ‘disputed’ mutations, some of which have high MICs on testing on solid medium with final reading at 6 weeks^12^.

Modifying MGIT either by reducing the critical concentration of rifampicin to 0.5 µg/ml and reading at 15 days, or using the standard critical concentration of 1 µg/ml with final reading at 21 days thus allow the improved detection of mutations D435Y, L452P and I491F, without misclassifying the WT strains. Reducing the concentration to 0.25 µg/ml at standard incubation time did not lead to satisfactory results, with only half as many correctly identified disputed mutations. Reduction of the critical concentration to 0.5 µg/ml and extended incubation period of 15 days would limit the increase of turnaround time compared to extended incubation at the standard concentration. A second critical concentration at half the standard was originally recommended not to miss such strains for the LJ proportion method^30^, although this has not been widely implemented.

We confirmed that commercially available genotypic DST methods miss disputed mutation I491F located outside the RRDR^21,39,48^ and found that the specificity of rifampicin rapid tests, whether phenotypic or genotypic, is good, although case reports of false resistance exist^49,50^.

The I491F mutation, which poses the greatest diagnostic challenge as it defies both phenotypic detection and detection by commercially available genotypic methods, drives outbreaks of undetected MDR-TB in Swaziland and South Africa^51,52^. MODS, and to a lesser extent NRA, having detected most of the I491F mutants, may be considered in these settings, besides MGIT modifications, although none of these approaches detect all I491F mutants, resulting in patients receiving multiple rounds of ineffective rifampicin based treatment in settings such as Swaziland and South Africa, direct *rpoB* sequencing on AFB-positive sputa may for now be the most accurate, albeit laborious approach. Alternatively, a genotypic method targeting the *rpoB* non-RRDR mutants, or even solely the I491F mutation, integrated within the diagnostic algorithm in these settings, may restore sensitivity to detect rifampicin resistance^53^.

Despite the same proportion of correctly detected RR strains, classic Xpert and Ultra showed varying degrees of resolution in their raw data. Classic Xpert raw data alone cannot determine the underlying ‘disputed’ mutation, whereas Ultra probe reaction and ∆Tm value can specifically identify ‘disputed’ and undisputed mutations in codon positions 430, 435, 450, and 452. This is exemplified by classic Xpert missed probe E capturing both ‘disputed’ mutation L452P and undisputed mutation S450L, whereas, Ultra probe rpoB4B distinguishes mutation L452P from S450L, which is identified by probe rpoB4A and associated ∆Tm value^39^. Ultra data also discriminate between mutations at the same codon, such as mutations D435Y and D435V, each characterized by a unique Ultra ∆Tm value linked to probe rpoB2^9^. Mutation L430P was likewise identified by distinct combination of probe rpoB1 and ∆Tm value. This improved resolution of Ultra to detect distinct (‘disputed’) mutations, although not a routine feature of the software, is very useful in interpreting discordant results between phenotypic and genotypic rifampicin resistance tests, as *rpoB* target sequencing is no longer necessary to resolve these^54^. Our observations support the key recommendation of Miotto and colleagues for genotypic DSTs to overrule MGIT results specifically when RRDR disputed mutations L430P, D435Y, and L452P are identified^15^.

## Conclusions

Our study provides more precise estimates of the impaired sensitivity of a wide variety of phenotypic tests to detect specific ‘disputed’ mutations within and outside the RRDR as rifampicin resistant. NRA and MODS were the most sensitive phenotypic DSTs to detect disputed mutations L430P, D435Y, L452P, and I491F, commercial MGIT and MYCOTB systems were the least sensitive. Compared with MGIT at standard pre-set time with 0.25 µg/ml ECOFF, we found a statistically significant increase in the ability of MGIT at extended incubation period of 15 and 21 days, with 0.5 µg/ml and 1 µg/ml ECOFF respectively, to resolve disputed mutants and WT strains, yet, it did not fully restore the sensitivity of MGIT. As predicted, the only mutant in our panel that escaped genotypic DST with current commercial assays was the I491F, for which better diagnostic tests are urgently needed.

## Supplementary information

Table S1. Results of the phenotypic and genotypic drug susceptibility tests
