# Supplementary material for: Variable ability of rapid tests to detect Mycobacterium tuberculosis rpoB mutations conferring phenotypically occult rifampicin resistance
Source: Sci Rep. 2019 Aug 14;9:11826. doi: 10.1038/s41598-019-48401-z (PMC6694172; doi:10.1038/s41598-019-48401-z)
Supplement: Supplementary file 1 — Table S1. Results of the phenotypic and genotypic drug susceptibility tests [file 41598_2019_48401_MOESM1_ESM.pdf]

Variable ability of rapid tests to detect *Mycobacterium tuberculosis rpoB* mutations conferring phenotypically occult rifampicin resistance

Gabriela Torrea, Kamela C. S. Ng, Armand Van Deun, Emmanuel André, Justine Kaisergruber, Willy Ssengooba, Christel Desmaretz, Siemon Gabriels, Michèle Driesen, Maren Diels, Sylvie Asnong, Kristina Fissette, Mourad Gumusboga, Leen Rigouts, Dissou Affolabi, Moses Joloba, Bouke C. de Jong

Table S1. Results of the phenotypic and genotypic drug susceptibility tests

ITM strain number	Geographic origin	*rpoB* sequencing result (*M. tuberculosis* numbering)	*rpoB* sequencing result (*E. coli* numbering)	LJ 40 µg/ml	MGIT (standard conditions) MIC (µg/ml)	MGIT (15 days ext.) MIC (µg/ml)	MGIT (21 days ext.) MIC (µg/ml)	MODS result	NRA result	REMA MIC (µg/ml)	Sensititre MIC (µg/ml)	classic Xpert	Xpert Ultra	LPA-Hain	LPA-Nipro
020333	BGD	L430P	L511P	S	≤0.125	0.25	1	S	S	≤0.125	≤0.12	R	R	S	R
041852	BGD	L430P	L511P	S	≤0.125	0.25	0.25	S	S	≤0.125	≤0.12	R	R	R	R
070065	BGD	L430P	L511P	R	≤0.125	0.5	1	R	S	≤0.125	0.25	R	R	S	R
050056	BGD	L430P	L511P	S	0.25	0.5	1	S	S	0.25	≤0.12	R	R	R	R
102773	BGD	L430P	L511P	S	0.25	0.5	1	S	S	≤0.125	≤0.12	R	R	R	R
970032	BGD	L430P	L511P	R	0.25	1	>1	S	R	0.25	0.5	R	R	R	R
031382	BGD	L430P	L511P	S	0.25	1	>1	S[1]	S	0.5	≤0.12	R	R	R	R
970858	BGD	L430P	L511P	R	1	>1	>1	R	R	>1	0.5	R	R	R	R
071723	BGD	L430P	L511P	S	>1	>1	>1	R	R	>1	4	R	R	R	R
070494	BGD	L430P	L511P	R	>1	>1	>1	R	R	1	>16	R	R	R	R
040934	BGD	L430P	L511P	S	ND	ND	ND	S	S	≤0.125	≤0.12	R	R	R	R
081321	BGD	D435Y	D516Y	S	≤0.125	0.5	>1	R	S	0.25	0.25	R	R	R	R
042325	BGD	D435Y	D516Y	R	0.25	1	>1	R	R	0.25	0.25	R	R	R	R
970118	BGD	D435Y	D516Y	S	0.25	0.25	1	S	S	0.5	≤0.12	R	R	R	R
111351	BGD	D435Y	D516Y	R	0.25	1	>1	R	S	0.3	≤0.12	R	R	R	R
052887	BGD	D435Y	D516Y	R	0.5	>1	>1	R	R	1	0.5	R	R	R	R
992158	BGD	D435Y	D516Y	R	0.5	>1	>1	R	R	0.5	0.5	R	R	R	R
083148	BGD	D435Y	D516Y	S	0.5	>1	>1	R	R	0.5	0.5	R	R	R	R
110628	BGD	D435Y	D516Y	R	0.5	>1	>1	R	R	0.5	2	R	R	R	R
072880	BGD	D435Y	D516Y	R	1	>1	>1	R	S	0.5	4	R	R	R	R
070260	BGD	D435Y	D516Y	R	ND	ND	ND	S	R	ND	>16	R	R	R	R

ITM strain number	Geog-raphic origin	*rpoB* sequen-cing result (*M. tubercu-losis* numbe-ring)	*rpoB* sequen-cing result (*E. coli* numbe-ring)	LJ 40 µg/ml	MGIT (stan-dard con-ditions) MIC (µg/ml)	MGIT (15 days ext.) MIC (µg/ml)	MGIT (21 days ext.) MIC (µg/ml)	MODS result	NRA result	REMA MIC (µg/ml)	Sensititre MIC (µg/ml)	classic Xpert	Xpert Ultra	LPA-Hain	LPA-Nipro
051911	BGD	L452P	L533P	R	≤0.125	>1	>1	R	S	0.25	0.5	R	R	R	R
061945	BGD	L452P	L533P	S	≤0.125	≤0.125	0.25	ND	R	ND	ND	R	R	R	R
063449	BGD	L452P	L533P	S	0.25	1	>1	R	R	≤0.125	0.5	R	R	R	R
052799	BGD	L452P	L533P	R	0.25	1	>1	S[1]	R	0.25	0.25	R	R	R	R
061066	BGD	L452P	L533P	R	0.25	1	1	S[2]	S	0.25	0.5	R	R	R	R
051380	BGD	L452P	L533P	R	0.25	0.5	0.5	R	R	>1	0.25	R	R	R	R
000811	BGD	L452P	L533P	S	0.25	0.5	>1	R	S	0.25	1	R	R	R	R
063693	BGD	L452P	L533P	R	0.5	1	>1	R	S	0.5	1	R	R	R	R
093251	BGD	L452P	L533P	R	ND	ND	ND	S[2]	S	≤0.125	≤0.12	R	R	R	R
950985	BGD	I491F	I572F	S	≤0.125	>1	>1	S	S	≤0.125	≤0.12	S	S	S	S
001329	BGD	I491F	I572F	R	0.25	1	>1	R	R	≤0.125	≤0.12	S	S	S	S
130654	BGD	I491F	I572F	R	0.25	1	>1	R	S	≤0.125	0.5	S	S	S	S
970576	BGD	I491F	I572F	S	0.25	0.5	0.5	S	S	≤0.125	≤0.12	S	S	S	S
072898	BGD	I491F	I572F	R	0.25	0.5	0.5	R	R	≤0.125	0.5	S	S	S	S
063667	BGD	I491F	I572F	R	0.25	1	>1	R	R	0.25	0.25	S	S	S	S
071340	BGD	I491F	I572F	R	0.5	>1	>1	R	R	1	0.5	S	S	S	S
130646	PAK	I491F	I572F	R	1	>1	>1	R	R	>1	2	S	S	S	S
112167	GEO	S450L	S531L	R	>1	>1	>1	R	R	>1	>16	R	R	R	R
120728	Bdesh	S450L	S531L	R	>1	>1	>1	R	R	>1	>16	R	R	R	R
121019	Bdesh	S450L	S531L	R	>1	>1	>1	R	R	>1	>16	R	R	R	R
121783	Bdesh	S450L	S531L	R	>1	>1	>1	R	R	>1	>16	R	R	R	R
132159	GEO	S450L	S531L	R	>1	>1	>1	R	R	>1	>16	R	R	R	R
130635	PAK	WT	WT	S	≤0.125	≤0.125	0.25	S	S	≤0.125	≤0.12	S	S	S	S

ITM strain number	Geog-raphic origin	*rpoB* sequen-cing result (*M. tubercu-losis* numbe-ring)	*rpoB* sequen-cing result (*E. coli* numbe-ring)	LJ 40 µg/ml	MGIT (stan-dard con-ditions) MIC (µg/ml)	MGIT (15 days ext.) MIC (µg/ml)	MGIT (21 days ext.) MIC (µg/ml)	MODS result	NRA result	REMA MIC (µg/ml)	Sensititre MIC (µg/ml)	classic Xpert	Xpert Ultra	LPA-Hain	LPA-Nipro
100936	GEO	WT	WT	S	≤0.125	0.25	0.5	S	S	≤0.125	≤0.12	S	S	S	S
130764	PAK	WT	WT	S	≤0.125	0.5	1	S	S	0.25	≤0.12	S	S	S	S
101161	ABK	WT	WT	S	≤0.125	0.25	0.5	S	S	≤0.125	≤0.12	S	S	S	S
101162	GEO	WT	WT	S	≤0.125	0.5	0.5	S	S	≤0.125	≤0.12	S	S	S	S
101177	ABK	WT	WT	S	≤0.125	0.5	0.5	S	S	≤0.125	≤0.12	S	S	S	S
130666	PAK	WT	WT	S	≤0.125	0.25	0.5	S	S	≤0.125	0.25	S	S	S	S
130719	PAK	WT	WT	S	≤0.125	0.5	0.5	S	S	≤0.125	0.25	S	S	S	S
132057	PAK	WT	WT	S	≤0.125	0.25	0.25	S	S	≤0.125	0.25	S	S	S	S
132066	PAK	WT	WT	S	≤0.125	0.5	1	S	S	0.25	≤0.12	S	S	S	S
991856	PER	WT	WT	S	≤0.125	≤0.125	≤0.125	R	ND	≤0.125	ND	S	S	S	S
101163	ABK	WT	WT	S	0.25	0.5	1	S	S	0.25	≤0.12	S	S	S	S
133221	PAK	WT	WT	S	0.25	0.5	0.5	ND	S	ND	0.5	S	S	S	S
083715[3]		H37Rv	H37Rv	S	0.25	0.5	1	S	ND	≤0.125	≤0.12	ND	ND	ND	ND
083715[4]		H37Rv	H37Rv	S	≤0.125	0.25	0.5	S	S	≤0.125	ND	ND	ND	ND	ND
083715[5]		H37Rv	H37Rv	S	≤0.125	≤0.125	≤0.125	S	S	≤0.125	ND	ND	ND	ND	ND
083715[6]		H37Rv	H37Rv	S	≤0.125	0.25	0.5	S	S	≤0.125	ND	ND	ND	ND	ND
083715[7]		H37Rv	H37Rv	S	0.25	0.5	0.5	S	S	≤0.125	ND	ND	ND	ND	ND

ABK, Abkhazia; BGD, Bangladesh; GEO, Georgia; PAK, Pakistan; PER, Peru; WT, wild-type; ND, no data; R, resistant; S, susceptible; RIF, rifampicin; RR, rifampicin resistant; MIC, Minimum Inhibitory Concentration; std., standard incubation time conditions; 15 days ext., 15 days extension of incubation time; [1], R extending incubation to 21 days; [2] R extending incubation to 23 days; [3,4,5,6,7], bacilli suspensions prepared from the same H37Rv strain tested at different times
